# An Analysis of Injury Trends and Disease Burden From Three Surveillance Hospitals in Urumqi From 2006 to 2018

**DOI:** 10.3389/fpubh.2022.915637

**Published:** 2022-07-22

**Authors:** Rong Zhang, Jing-Xuan Sun, Ying-Zhen Guo, Lai-Xin Liu, Fuerhati Wushouer, Yan Dong, Ping Fang, Xiamusiye Muyiduli, Zhen-Guo Gao, Jiang-Hong Dai, Ming-Jian Ni

**Affiliations:** ^1^Post-doctoral Mobile Research Station of Public Health and Preventive Medicine, School of Public Health, Xinjiang Medical University, Urumqi, China; ^2^Post-doctoral Research Station, Xinjiang Uygur Autonomous Region Center for Disease Control and Prevention, Urumqi, China; ^3^Department for Chronic and Non-communicable Disease Control and Prevention, Tianshan District Center for Disease Control and Prevention, Urumqi, China

**Keywords:** surveillance, injury, hospital-based, distribution, disease burden

## Abstract

**Objective:**

To investigate injury trends, injury distribution, and disease burden from three surveillance hospitals in Urumqi from 2006 to 2018.

**Method:**

Injury data from the National Injury Surveillance System (NISS) from three hospitals in Urumqi (2006 to 2018) were collected to analyze changes in the characteristics of outpatient injury cases. Years of potential life lost (YPLL) were calculated to determine the disease burden of the injury cases.

**Results:**

A total of 161,400 injury cases were recorded over 13 years, and the average age of the patient seeking medical attention was 32.4 years old. Male patients outnumbered female patients with a ratio of 1.6:1, but the proportion of female patients was greater after 45 years of age. The highest number of cases occurred in patients 15–29 years of age, accounting for 26.8% of all injury cases. Injury in females occurred most frequently in the home. A total of 41.4% of injury cases occurred while doing housework. The top three causes of injury were falls (49.7%), blunt force of an object, (13.7%), and motor vehicle accidents (MVA) (13.5%). Years of potential life lost from injury accounted for 7.39% of the total YPLL in the three hospitals.

**Conclusion:**

Males should be targeted for injury prevention and intervention in Urumqi. The prevention of falls, blunt force of objects, and MVA should be made a priority. Injury prevention strategies and targeted projects should be developed to reduce the disease burden of injury.

## Introduction

Injury is the third leading cause of death worldwide and is a major cause of death and disability in individuals aged 5–44 years. Globally, injuries cause >5 million deaths annually—a similar number to those from HIV/AIDS, tuberculosis and malaria combined (1). More young people are affected by injury than the elderly, leading to more years of life lost (YLL) than those lost due to chronic and infectious diseases (2). In 2010, the crude death rate of injury in China was 59.34/100,000, and the standardized death rate was 61.87/100,000. The YLL due to injury was 31.7593 million person years, the years lived with disability (YLD) due to injury was 9.0447 million person years, and the disability-adjusted life years (DALYs) due to injury was 40.8040 million person years (3).

The patterns of injury mortality differ between countries and are related to essential demographic characteristics, such as economic income, customs, and lifestyle (4). As a developing country, China's rapid economic growth has been accompanied by dramatic changes in lifestyle, modes of transportation, work environments, and other factors, which have caused an unexpected increase in injury rates. Injury has become the fourth leading cause of death in China (5). Xinjiang, the Xinjiang Uygur Autonomous Region, located in northwest China, is not an economically fast-growing province; nevertheless, it has experienced economic and lifestyle changes in recent years. In addition to changes in social development and lifestyle, the pattern of injury mortality has increased. Injury is the fifth leading cause of death in Xinjiang (6).

Two hundred million injury events occur each year, leading to 14 million hospitalizations and 1 million disabilities per year (7). Injuries affect the health and welfare of all age groups because of potential disability, medical costs, and premature death (5). The National Injury Surveillance System (NISS) was established in 2006 and is a hospital-based surveillance system. Three hospitals from Urumqi were selected as the surveillance sites for the system (8). We conducted this study using the NISS data from 2006 to 2018 to describe the characteristics and disease burden of injury in surveillance hospitals in Urumqi and to provide basic data for the development of a series of preventive countermeasures in Xinjiang. This data demonstrates the incidence of injury in outpatient and emergency centers in Xinjiang. This is the most complete and best injury database in Xinjiang from 2006 to 2018, and the first time to show situation of injuries in outpatient and emergency in Urumqi by NISS.

## Methods

### Ethics Approval

Three hospitals of Urumqi from 2006 to 2018 were extracted to analyze changes in the characteristics of outpatient visits due to injury. The study was approved by the ethical review committee for the National Center for Chronic and Non-communicable Disease Control and Prevention (NCNCD), and the Chinese Center for Disease Control and Prevention (CDC), Beijing, People's Republic of China (No. 201310).

### Surveillance Hospitals and Case Definitions

Three hospitals in Xinjiang served as the surveillance sites, including the People's Hospital of Xinjiang Uygur Autonomous Region, the Sixth Teaching Hospital of Xinjiang Medical University, and the First People's Hospital of Urumqi.

An injury case was defined as the first presentation of a patient in the emergency room or other clinical ward of one of the surveillance hospitals that receive outpatients. Patients who sought medical attention in other medical institutions or in the same hospital for the same (repeat visit) injury event were excluded. An injury is defined by the World Health Organization as “physical damage to the human body due to acute exposure to energy or a lack of a vital element” (9).

### Data Collection and Quality Control

A standardized data collection form was developed for patients by the NCNCD. It contained questions regarding basic patient information, basic injury information, and clinical injury information. Face-to-face training sessions were held with item-by-item explanations of all data elements at each hospitals.

Doctors and/or nurses recorded information in the NISS surveillance form based on the information in the patients' medical records. After the forms were filled out, they were collected and stored by each hospital's prevention and health protection department and submitted monthly to the CDC. In the Tianshan district CDC office, the data were entered into a database using a uniform and specialized software program, the National Injury Information System, developed by the NCNCD. The database were reported to the Xinjiang CDC and then quarterly to the central office of the NCNCD.

### Statistical Analysis

Study data were analyzed using Statistical Product and Service Solutions (SPSS) for Windows, Version 13.0. (SPSS Inc., Chicago, IL, USA.). Outliners values were handled as missing data. Descriptive statistics such as numerical variables are presented as means and standard deviations, while categorical variables are presented as frequencies and percentages.

## Results

### Trends in the Proportion of Injury Cases From 2006 to 2018

From 2006 to 2018, a total of 161,400 injury cases were recorded from three hospitals in Urumqi, with 1,035 cases recorded per month for all three hospitals. In total, 62.0% (100,082/161,400) of cases occurred in males, and 38.0% (61,218/161,400) of cases occurred in females. The mean age of the patients was 32.4 ± 20.4 years old. Males outnumbered females with a ratio of 1.6:1. Most of the injury cases reported in the NISS occurred in patients 15–64 years of age. A total of 74.1% (119,559/161,400) of the patients were local city/county residents. Students, commercial/service personnel, and professionals were the top three occupations. Junior middle school (35,271/161,400) and primary school (34,513/161,400) were the top two education levels. The annual number of injury cases reported by the NISS of the three hospitals increased from 3,317 in 2006 to 22,880 in 2018. In each year, males outnumbered females, especially after 2010.

### Age Groups of Injury Cases

**Figure 1:**
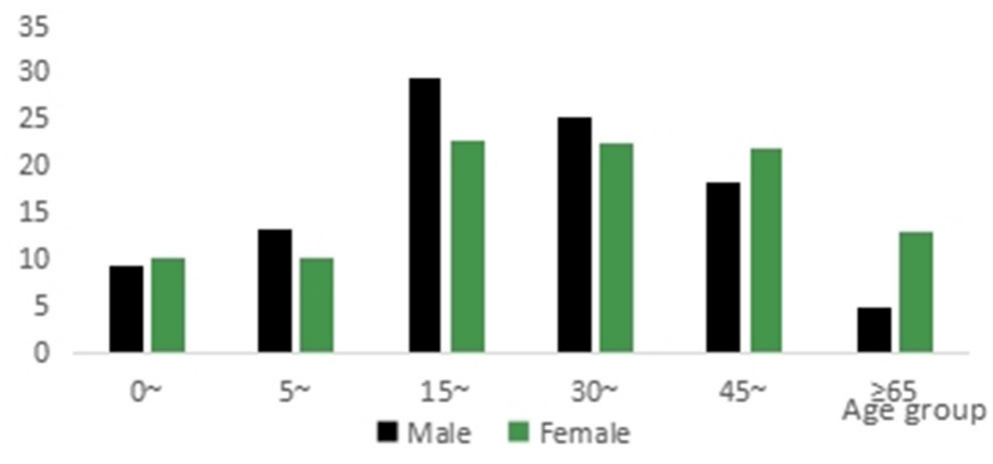
Injury cases by age group and gender.

The top three age groups that presented with injury were 15–29 (26.8%), 30–44 (24.3%), and 45–64 (19.6%) years of age, accounting for 70.7% of all cases (Table 1). The age trends of male and female injury cases were the same. There were more female injury cases than male injury cases in individuals aged 0–4 years of age and 45 years of age and older (Figure 1).

**Table 1:** Injury cases by age group and gender.

**Age group**	**Male**		**Female**		**Total**	
	***N***	**%**	***N***	**%**	***N***	**%**
0–5	9,240	9.2	6,102	10.0	15,342	9.5
5–15	13,151	13.1	6,166	10.1	19,317	12.0
15–30	29,276	29.3	13,934	22.7	43,210	26.8
30–45	25,360	25.3	13,795	22.5	39,155	24.3
45–65	18,162	18.1	13,418	21.9	31,580	19.6
≥65	4,880	4.9	7,892	12.9	12,772	7.9
Total	100,069	100.0	61,307	100.0	161,376	100.0

*N, Number*.

### Location of Injury Cases

The three leading locations where injury occurred were public residential institutions (26.5%), homes (26.0%), and roads/streets (22.5%). The proportion of injuries sustained in the home was higher in females (34.4%) than in males (20.9%), and the proportion of injuries sustained from sports and athletics was higher in males (10.0%) than in females (1.8%) (Table 2).

**Table 2:** The proportions of injuries sustained at different places by gender.

**Injury place**	**Male**		**Female**		**Total**	
	***N***	**%**	***N***	**%**	***N***	**%**
Home	20,877	20.9	21,084	34.4	41,961	26.0
Public residential institution	27,475	27.5	15,301	25.0	42,776	26.5
School and public areas	9,436	9.4	4,210	6.9	13,646	8.5
Sports and athletics areas	2,520	2.5	887	1.4	3,407	2.1
Road/street	21,351	21.3	15,000	24.5	36,351	22.5
Commercial and service areas	7,055	7.0	3,041	5.0	10,096	6.3
Industrial and construction areas	10,013	10.0	1,098	1.8	11,111	6.9
Farm/farmland	608	0.6	190	0.3	798	0.5
Other	384	0.4	277	0.5	661	0.4
Unclear	361	0.4	230	0.4	591	0.4
Total	100,080	100.0	61,318	100.0	161,398	100.0

*N, Number*.

### Activity at the Time of Injury

For the total population, the top three activities at the time of injury were: performing housework, recreational activities, and paid work. For males, activities in order of the number of injuries were housework, recreational activities, and paid work. For females, activities in order of the number of injuries were housework, recreational activities, and walking. Injuries were more likely to occur for males while doing paid work and educational activities and more likely to occur for females while doing housework and recreational activities (Table 3).

**Table 3:** The proportion of injuries sustained during different activities by gender.

**Injury activity**	**Male**		**Female**		**Total**	
	***N***	**%**	***N***	**%**	***N***	**%**
Paid work	11,093	11.1	3,010	4.9	14,103	8.7
Housework	39,995	40.0	26,862	43.8	66,857	41.4
Education	8,050	8.0	2,130	3.5	10,180	6.3
Sports activities	5,018	5.0	3,247	5.3	8,265	5.1
Recreational activities	21,229	21.2	13,699	22.3	34,928	21.6
Vital movement	6,027	6.0	4,590	7.5	10,617	6.6
Driving/riding in a vehicle	2,871	2.9	2,016	3.3	4,887	3.0
Walking	5,387	5.4	5,449	8.9	10,836	6.7
Other	248	0.2	199	0.3	447	0.3
Unclear	161	0.2	114	0.2	275	0.2
Total	100,079	100.0	61,316	100.0	161,395	100.0

*N, Number*.

### Cause of Injury

The three leading causes of injury were falls, blunt force of an object, and motor vehicle accidents (MVA). Injuries were more likely to be caused by blunt force of an object or knife/sharp object in males, and more likely to be caused by falls or burns in females (Table 4).

**Table 4:** The proportions of different injury causes by gender.

**Injury cause**	**Male**		**Female**		**Total**	
	***N***	**%**	***N***	**%**	***N***	**%**
Motor vehicle accident	13,034	13.0	8,796	14.3	21,830	13.5
Non-motor vehicle accident	1,533	1.5	786	1.3	2,319	1.4
Fall	46,760	46.7	33,531	54.7	80,291	49.7
Blunt for by object	16,166	16.2	5,924	9.7	22,090	13.7
Firearm	85	0.1	38	0.1	123	0.1
Knife/sharp object	11,224	11.2	4,353	7.1	15,577	9.7
Burn	1,499	1.5	1,377	2.2	2,876	1.8
Suffocation/hanging	45	0.0	21	0.0	66	0.0
Drowning	9	0.0	5	0.0	14	0.0
Poisoning	1,697	1.7	1,933	3.2	3,630	2.2
Animal contact	1,136	1.1	823	1.3	1,959	1.2
Sexual assault	5	0.0	5	0.0	10	0.0
Other	6,561	6.6	3,477	5.7	10,038	6.2
Unclear	325	0.3	246	0.4	571	0.4
Total	100,079	100.0	61,315	100.0	161,394	100.0

*N, Number*.

### Injury Intention

Falls were the most common cause of unintentional injury. Injury with a blunt object was the most common cause of an assault injury. A knife/sharp object injury was the most common cause of an intentional self-harm injury in males, and poisoning was the most common cause of an intentional self-harm injury in females (Table 5).

**Table 5:** The proportions of injury cases by cause and intent (%).

	**Unintentional**			**Intentional self-harm**			**Assault**			**Undetermined**			**Total**		
	**Male**	**Female**	**Total**	**Male**	**Female**	**Total**	**Male**	**Female**	**Total**	**Male**	**Female**	**Total**	**Male**	**Female**	**Total**
Fall	53.2	60.1	55.9	7.8	4.3	5.7	2.2	3.0	2.4	22.1	15.3	19.5	46.7	54.7	49.7
Vehicle accident	16.5	17.2	16.8	2.6	1.5	1.9	0.4	0.6	0.5	23.9	14.7	20.4	14.5	15.6	14.9
Blunt force by object	11.9	6.7	9.9	7.1	1.4	3.6	48.7	48.3	48.6	8.4	5.6	7.4	16.2	9.7	13.7
Knife/sharp object	9.9	6.1	8.4	44.4	29.9	35.5	19.0	13.1	17.4	8.8	8.5	8.7	11.2	7.1	9.7
Animal contact	1.2	1.4	1.3	0.1	0.2	0.1	0.8	1.0	0.9	2.5	2.8	2.6	1.1	1.3	1.2
Burn	1.7	2.5	2.0	0.1	0.2	0.1	0.1	0.1	0.1	0.4	0.0	0.2	1.5	2.2	1.8
Poisoning	1.6	2.0	1.8	27.7	55.3	44.7	0.3	1.8	0.7	12.6	25.4	17.5	1.7	3.2	2.2
Suffocation/hanging	0.0	0.0	0.0	0.3	0.0	0.1	0.0	0.0	0.0	0.4	0.0	0.2	0.0	0.0	0.0
Firearm	0.1	0.0	0.1	0.1	0.0	0.0	0.2	0.4	0.2	0.0	0.6	0.2	0.1	0.1	0.1
Drowning	0.0	0.0	0.0	0.0	0.1	0.0	0.0	0.0	0.0	0.0	0.0	0.0	0.0	0.0	0.0
Sexual assault	0.0	0.0	0.0	0.1	0.0	0.0	0.0	0.1	0.0	0.0	0.0	0.0	0.0	0.0	0.0
Other	3.7	3.6	3.7	8.3	4.6	6.0	27.7	31.3	28.7	8.1	4.5	6.7	6.6	5.7	6.2
Unknown	0.3	0.3	0.3	1.3	2.6	2.1	0.4	0.4	0.4	13.0	22.6	16.7	0.3	0.4	0.4
Total	100.0	100.0	100.0	100.0	100.0	100.0	100.0	100.0	100.0	100.0	100.0	100.0	100.0	100.0	100.0

### Disease Burden of Injury

The disease burden analysis in the three surveillance hospitals was calculated using YPLL up to 70 years based on the Chinese National Disease Surveillance Points data from 2018. In total, the estimated YPLL was 8,800. Injuries accounted for 7.39% of the total YPLL from 2018.

## Discussion

A total of 161,400 injury cases were recorded over 13 years. Male patients outnumbered female patients by 1.6:1, but the proportion of female patients was greater after 45 years of age. The 15–29 years of age group accounted for 26.8% of the total injuries, which was the highest percentage according to age group. Injury occurred most frequently in females while in the home. Years of potential loss of life from injuries accounted for 7.39% of the total YPLL in the three hospitals.

We found that there were more injuries in male patients than female patients in each year from 2006 to 2018, and the proportion of females was greater in patients 45 years of age and older and 0–4 years of age, consistent with previous national reports (10). Our study revealed that more injuries occurred in females when doing housework than in males. Males were more likely than females to be injured in industrial and construction areas while doing paid work. This may be because males have a wider range of activities and are more likely to engage in risky behaviors, have a high-risk occupation, and have greater exposure to risk factors than females, leading to a higher overall injury rate. After 45 years of age, there is a decline in physiological function, psychological function, and social adaptability caused by aging. At this age, females become the high-risk group. More psychological counseling and care should be provided for them.

The main cause of intentional self-harm in females was poisoning. This was similar to national findings (11). Interventions for self-inflicted injury/attempted suicide should be strengthened, including control programs for pesticides and psychotropic drugs. Psychological counseling, psychological crisis intervention, enhanced care and social support should be provided for females. A person who has previously attempted suicide has a higher risk of suicide than those who have never attempted suicide, with 27% of suicide victims in China having had a history of attempted suicide (12). Preventive measures should be implemented during hospitalization for those who have attempted suicide.

Falls were the main type of injury in the three surveillance hospitals, accounting for 43.7% of the injuries from 2006 to 2018. This result was consistent with other study findings (13). This may be the result of an aging population. Fall prevention programs targeting elderly individuals and children should be prioritized (14). Attention should be paid to improving the environmental risk factors in homes and public living places.

Death due to disease has decreased, and injury is now considered “the last major plague of the young” in developed countries. However, as a developing country, the economy of China has grown rapidly, resulting in changes in lifestyle, nutrition, transportation, and other factors that have caused an unexpected increase in injury. By 2010, ~796,000 people had died from injuries, with the age-standardized mortality rate reaching 57/100,000 people. The mortality rates due to injury showed a significant decline between 1990 and 2010, and although they were lower than those of chronic non-communicable and infectious diseases in China, injury prevention and mitigation were in need of development (15, 16). Years of potential loss of life from injury accounted for 7.39% of the total YPLL in the three hospitals. The burden of injury should be widely recognized. Prevention strategies and targeted projects should be developed to reduce the disease burden of injury.

The NISS in Xinjiang is an important part of the disease monitoring system, and it is the most reliable source of injury data to support the development of epidemiological studies on injury prevention and control. The occurrence of some injuries is inevitable, but many can be prevented. Injury prevention and control can be accomplished by social system engineering based on the epidemiological characteristics of injuries, such as the causes and characteristics of injuries. Effective strategies and measures for injury prevention and mitigation are available, such as fencing around swimming pools to prevent falls and drownings, and safety legislation and government regulations that have been issued and implemented to prevent traffic accidents. For example, the Road Traffic Safety Law established regulations for road traffic safety in 2004 and additional regulations for drunk driving in 2011 (17).

Some limitations to our study should be noted. First, it was a hospital-based surveillance study of data from the NISS. Second, only the data from three hospitals in Urumqi were included. Generalization of our results should be considered with caution. Third, only patients with an injury presenting to an emergency room, outpatient department, or other clinical department in the three surveillance hospitals were included in the analysis, and deaths that occurred outside of the hospitals were not recorded in the NISS, which may have resulted in the underestimation of injury-related deaths in this study. Our analysis provided data and indicators for understanding injuries and their health-related problems in Xinjiang. It also called for more attention and support for injury prevention and control in this area of a rapidly developing country as large as China.

## Conclusions

Injury data from the National Injury Surveillance System (NISS) from three hospitals in Urumqi (2006 to 2018) were collected. There were more male patients with injury than female in each year from 2006 to 2018, but the proportion of female patients was greater than that of male patients after 45 years of age. The percentage of injury in the 15–29 years of age group was the highest. Most of the injuries in females occurred in the home while doing housework. Males were more likely to be injured in industrial and construction areas while doing paid work. Falls were the major cause of injury. Men should be protected at work, women who do housework should be protected at home. More attention should be paid to 15–29 years people.

## Data Availability Statement

The original contributions presented in the study are included in the article/supplementary material, further inquiries can be directed to the corresponding author/s.

## Ethics Statement

The studies involving human participants were reviewed and approved by Ethical Review Committee of the National Center for Chronic and Non-communicable Disease Control and Prevention (NCNCD), Chinese Center for Disease Control and Prevention, Beijing, People's Republic of China (No. 201310). The patients/participants provided their written informed consent to participate in this study.

## Author Contributions

J-HD and M-JN conceived the study idea. Y-ZG, L-XL, FW, YD, PF, and Z-GG acquired the data. RZ conducted, analyzed, and interpreted the results. RZ, J-XS, XM, J-HD, and M-JN were involved in drafting the manuscript and made critical revisions to the manuscript for important intellectual content. All authors read and approved the manuscript.

## Funding

This research was supported by the Natural Science Foundation of Xinjiang Uygur Autonomous Region (Grant Number 2017D01B38).

## Conflict of Interest

The authors declare that the research was conducted in the absence of any commercial or financial relationships that could be construed as a potential conflict of interest.

## Publisher's Note

All claims expressed in this article are solely those of the authors and do not necessarily represent those of their affiliated organizations, or those of the publisher, the editors and the reviewers. Any product that may be evaluated in this article, or claim that may be made by its manufacturer, is not guaranteed or endorsed by the publisher.
